# Supplementary material for: Complete map of SARS-CoV-2 RBD mutations that escape the monoclonal antibody LY-CoV555 and its cocktail with LY-CoV016
Source: Cell Rep Med. 2021 Apr 5;2(4):100255. doi: 10.1016/j.xcrm.2021.100255 (PMC8020059; doi:10.1016/j.xcrm.2021.100255)
Supplement: Document S1. Figures S1 and S2 [file mmc1.pdf]

Cell Reports Medicine, Volume *2*

## Supplemental information

# Complete map of SARS-CoV-2 RBD mutations that escape the monoclonal antibody LY-CoV555 and its cocktail with LY-CoV016

**Tyler N. Starr, Allison J. Greaney, Adam S. Dingens, and Jesse D. Bloom**

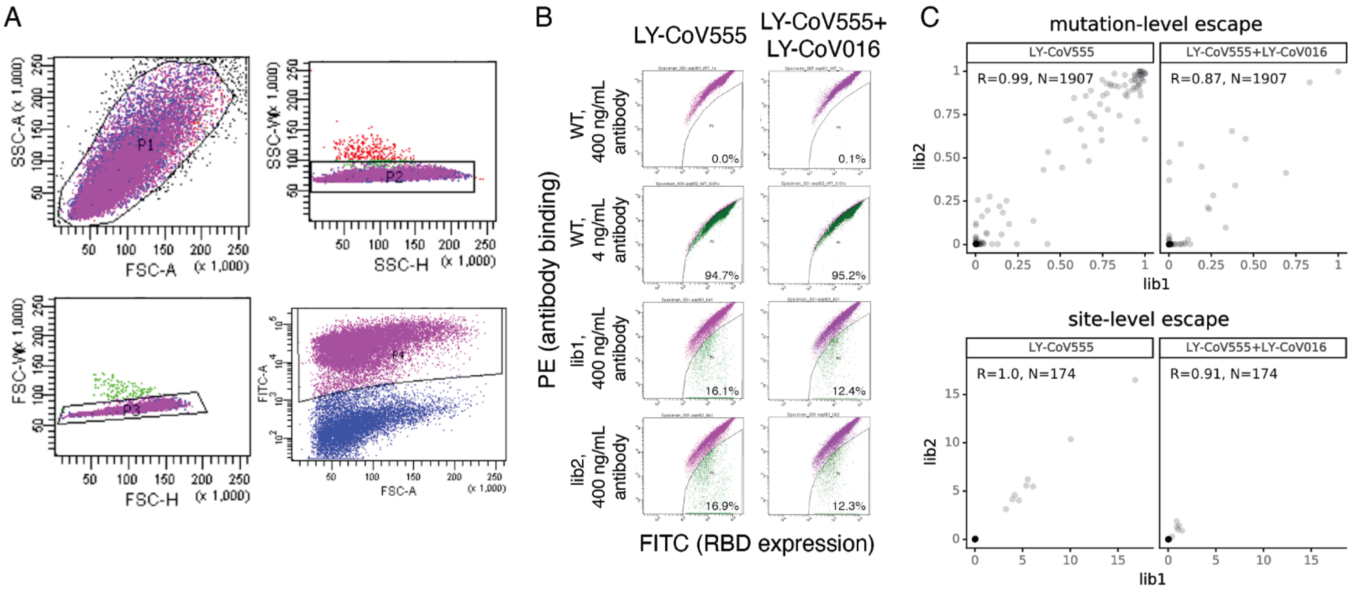


**Figure S1. Experimental approach for antibody escape mapping. Related to Figure 1.** We use fluorescence-activated cell sorting to select yeast mutants that escape antibody binding. We use libraries described by Starr et al. [S1] that contain virtually all RBD amino acid mutations, which were previously sorted to select for those compatible with human ACE2 binding as described by Greaney et al. [S2]. (A) Initial FACS gates are drawn to select single yeast cells (SSC/FSC, SSC-W/SSC-H, and FSC-H/FSC-W) that properly express RBD (FITC/FSC). (B) Antibody-escape bins are drawn to capture 95% of RBD$^{+}$ cells expressing the unmutated SARS-CoV-2 RBD labeled at 4 ng/mL, indicating 100x reduced binding compared to the 400 ng/mL library selections. The fraction of cells in each population that fall into this "antibody-escape" bin is labeled in each FACS plot. (C) Correlation between independent library duplicates at the level of per-mutation escape fractions (top) or site-level total escape (sum of all mutations at site, bottom). We report the average of these duplicate measurements in the text, given in Data S1.

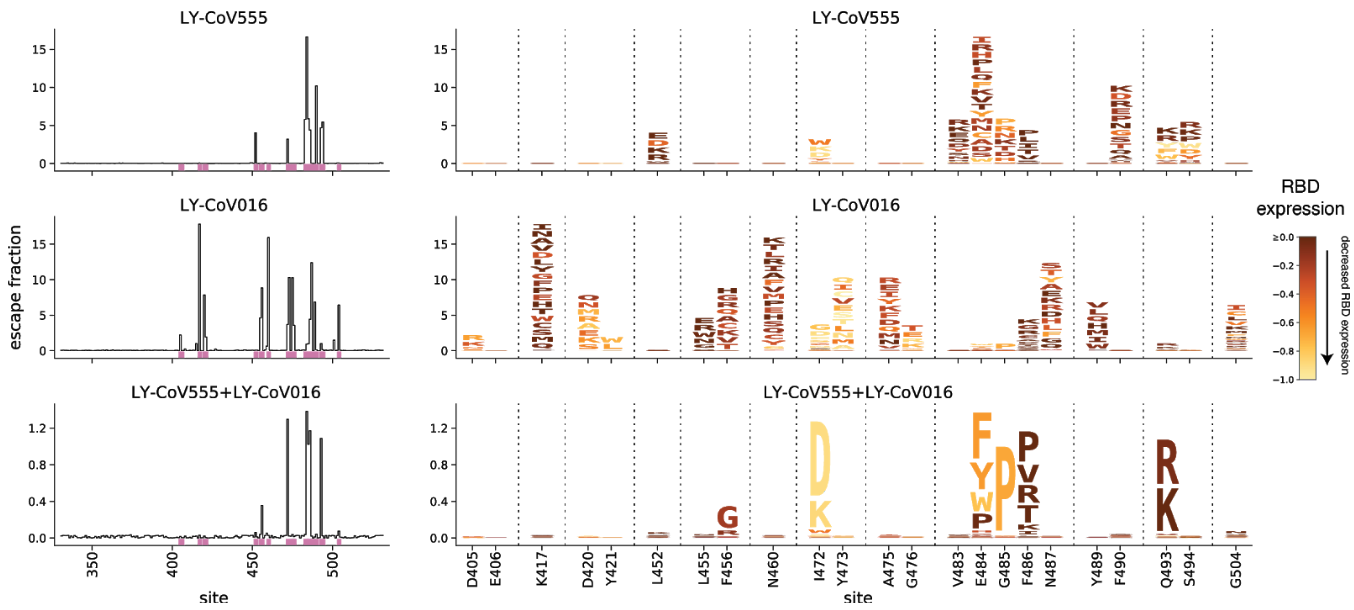


**Figure S2. Logoplots colored by mutation effects on expression. Related to Figure 1.** Line and logoplots are identical to Fig. 1A, except mutations are colored by their effects on the yeast surface expression level of folded RBD, as measured in our previous deep mutational scanning experiment [S1].

**Supplemental References**

[S1] Starr TN, Greaney AJ, Hilton SK, et al. Deep Mutational Scanning of SARS-CoV-2 Receptor Binding Domain Reveals Constraints on Folding and ACE2 Binding. *Cell* 2020; 182: 1295–1310.e20.

[S2] Greaney AJ, Starr TN, Gilchuk P, et al. Complete Mapping of Mutations to the SARS-CoV-2 Spike Receptor-Binding Domain that Escape Antibody Recognition. *Cell Host Microbe* 2021; 29: 44–57.e9.
