# Supplementary material for: Complete map of SARS-CoV-2 RBD mutations that escape the monoclonal antibody LY-CoV555 and its cocktail with LY-CoV016
Source: Cell Rep Med. 2021 Apr 5;2(4):100255. doi: 10.1016/j.xcrm.2021.100255 (PMC8020059; doi:10.1016/j.xcrm.2021.100255)
Supplement: Document S2. Article plus supplemental information [file mmc3.pdf]

Cell Reports
Medicine

Report

Complete map of SARS-CoV-2 RBD mutations that escape the monoclonal antibody LY-CoV555 and its cocktail with LY-CoV016

Graphical abstract

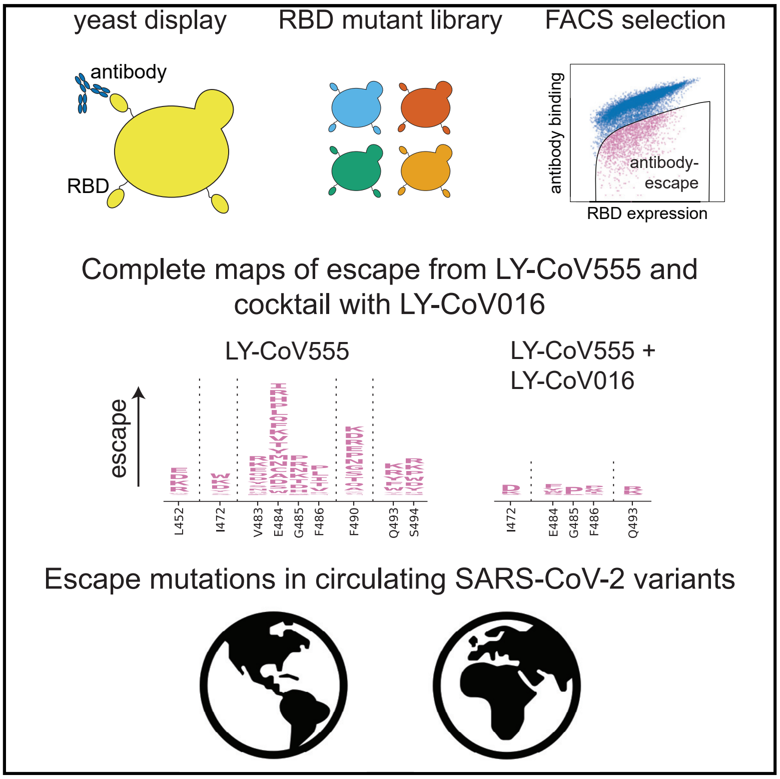

Authors

Tyler N. Starr, Allison J. Greaney, Adam S. Dingens, Jesse D. Bloom

Correspondence

jbloom@fredhutch.org

In brief

Starr et al. report a complete map of all of the SARS-CoV-2 mutations that escape binding by the LY-CoV555 (bamlanivimab) antibody and its cocktail with LY-CoV016. These maps highlight high-frequency mutations that may affect LY-CoV555 efficacy and enable immediate interpretation of the effects of future viral variation on this antibody.

Highlights

- Map of all SARS-CoV-2 mutations that escape LY-CoV555 (bamlanivimab) antibody binding
- LY-CoV555 is sensitive to mutations E484K and L452R in variants of concern
- Single mutations and circulating combinations escape the LY-CoV555/LY-CoV016 cocktail
- Therapeutic antibodies that target subdominant epitopes are needed

Starr et al., 2021, Cell Reports Medicine 2, 100255
April 20, 2021 © 2021 The Author(s).
https://doi.org/10.1016/j.xcrm.2021.100255

CellPress

Cell Reports Medicine

CellPress
OPEN ACCESS

Report

Complete map of SARS-CoV-2 RBD mutations that escape the monoclonal antibody LY-CoV555 and its cocktail with LY-CoV016

Tyler N. Starr,[1] Allison J. Greaney,[1,2,3] Adam S. Dingens,[1,5] and Jesse D. Bloom[1,2,4,6,*]
[1]Basic Sciences and Computational Biology, Fred Hutchinson Cancer Research Center, Seattle, WA 98109, USA
[2]Department of Genome Sciences, University of Washington, Seattle, WA 98109, USA
[3]Medical Scientist Training Program, University of Washington, Seattle, WA 98109, USA
[4]Howard Hughes Medical Institute, Seattle, WA 98109, USA
[5]Present address: Shape Therapeutics, Seattle, WA 98109, USA
[6]Lead contact
*Correspondence: jbloom@fredhutch.org
https://doi.org/10.1016/j.xcrm.2021.100255

SUMMARY

Monoclonal antibodies and antibody cocktails are a promising therapeutic and prophylaxis for coronavirus disease 2019 (COVID-19). However, ongoing evolution of severe acute respiratory syndrome-coronavirus-2 (SARS-CoV-2) can render monoclonal antibodies ineffective. Here, we completely map all of the mutations to the SARS-CoV-2 spike receptor-binding domain (RBD) that escape binding by a leading monoclonal antibody, LY-CoV555, and its cocktail combination with LY-CoV016. Individual mutations that escape binding by each antibody are combined in the circulating B.1.351 and P.1 SARS-CoV-2 lineages (E484K escapes LY-CoV555, K417N/T escapes LY-CoV016). In addition, the L452R mutation in the B.1.429 lineage escapes LY-CoV555. Furthermore, we identify single amino acid changes that escape the combined LY-CoV555+LY-CoV016 cocktail. We suggest that future efforts diversify the epitopes targeted by antibodies and antibody cocktails to make them more resilient to the antigenic evolution of SARS-CoV-2.

INTRODUCTION

Monoclonal antibodies have been rapidly developed for the treatment and prophylaxis for coronavirus disease 2019 (COVID-19) where they have shown promise in humans[1,2] and animal models.[3–7] One leading antibody is LY-CoV555 (bamlanivimab),[4] which has an emergency use authorization (EUA) for the therapeutic treatment of COVID-19.[8] An EUA was also recently granted for the administration of LY-CoV555 as a cocktail with another antibody, LY-CoV016 (also known as etesevimab).[9]

A key question is whether the ongoing evolution of severe acute respiratory syndrome-coronavirus-2 (SARS-CoV-2) will lead to escape from these antibodies. This question has taken on growing importance with the recent emergence of SARS-CoV-2 lineages containing mutations in the spike receptor-binding domain (RBD),[10–13] the target of the most clinically advanced antibodies, including LY-CoV555 and LY-CoV016. A flurry of recent studies has addressed this question by characterizing the antigenic effects of the mutations in these emerging lineages. Unfortunately, some of the mutations in emerging lineages reduce binding and neutralization by some key antibodies in clinical development, including LY-CoV555 and LY-CoV016.[14–17]

To enable more comprehensive and prospective assessment of the effects of viral mutations, we recently developed a method to completely map how all single amino acid mutations in the SARS-CoV-2 RBD affect antibody binding.[15,18,19] These maps enable immediate interpretation of the consequences of new mutations and systematic comparison of escape mutations across antibodies.

Here, we prospectively map how all mutations to the RBD affect binding by LY-CoV555 alone and in a cocktail with LY-CoV016. (We previously reported how all mutations affect binding by LY-CoV016 alone.[15]) Binding by LY-CoV555 is escaped by mutations within and near the RBD "receptor-binding ridge," including by mutations at sites L452 and E484, which are present in emerging viral lineages. Furthermore, the LY-CoV555+LY-CoV016 cocktail is escaped by the specific combinations of mutations at K417 and E484 found in the B.1.351 and P.1 lineages. Finally, we show that several individual amino acid mutations are capable of escaping the combined LY-CoV555+LY-CoV016 cocktail.

RESULTS

We applied a previously described deep mutational scanning approach to comprehensively map mutations in the SARS-CoV-2 RBD that escape binding from antibodies.[15,18,19] Briefly, this method involves displaying nearly all of the amino acid mutants of the SARS-CoV-2 RBD on the surface of yeast,[20] incubating the yeast with an antibody or antibody cocktail, using

© 2021 The Author(s).
This is an open access article under the CC BY license (http://creativecommons.org/licenses/by/4.0/).

A

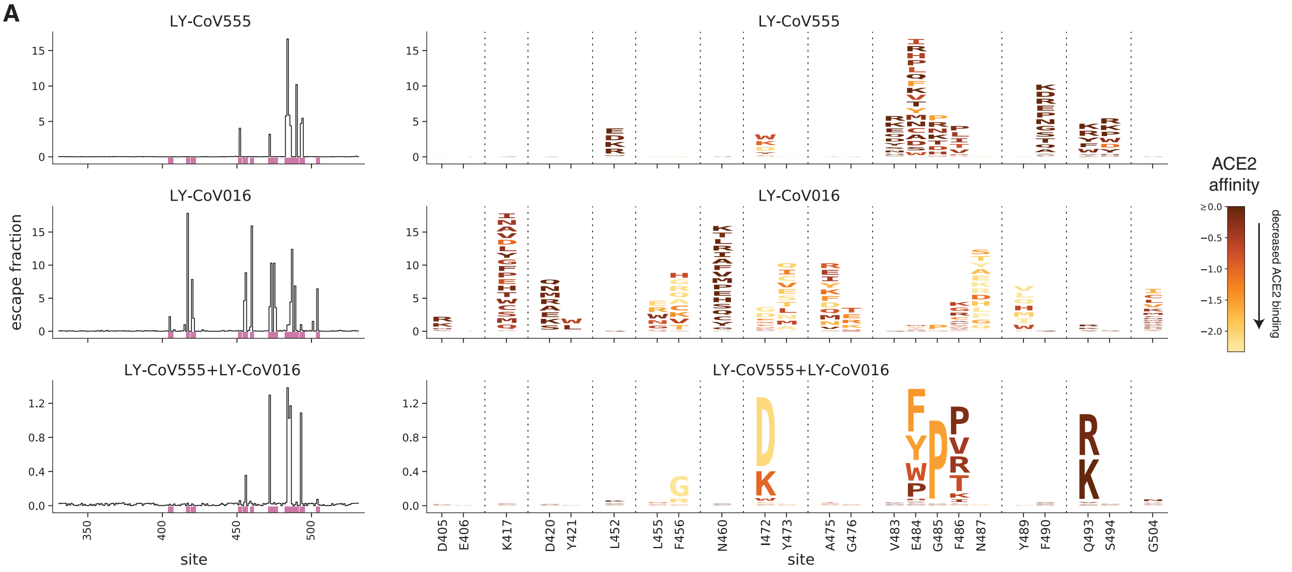

B

mutation	LY-CoV555 fold change IC_{50}*	LY-CoV016 fold change IC_{50}*
K417N	0.12	>1000
E484K	>1000	3.5
N501Y	1.0	2.6

* neutralization assays from Wang et al. [14]

Figure 1. Comprehensive escape maps for LY-CoV555, LY-CoV016, and a 1:1 cocktail of the 2 antibodies

(A) Newly described escape maps for LY-CoV555 and LY-CoV555+LY-CoV016 cocktail, alongside our previously reported escape map for LY-CoV016.[15] Line plots at left show the total escape (sum of per-mutation escape fractions) at each RBD site. Sites indicated by pink lines on the x axis are then shown in magnified form in the logoplots at right. In these logoplots, the height of each letter indicates the escape fraction for that mutation (larger letters mean stronger escape from antibody binding). Letters are colored by how mutations affect ACE2 binding affinity (scale bar, bottom right), as measured in our prior deep mutational scan.[20] See Figure S2 for escape maps colored by mutation effects on folded RBD expression and Data S1 for raw data. Note that the y axis is scaled differently for each antibody/cocktail. The sites shown in the logoplots are where mutations have an appreciable effect on either antibody, as well as site 406 (which is an escape mutation from the REGN-COV2 cocktail[15]).

(B) Literature measurements of the effects of K417N, E484K, and N501Y on neutralization by LY-CoV555 and LY-CoV016.[14] These measurements validate our maps, which suggest that K417N specifically escapes LY-CoV016, E484K specifically escapes LY-CoV555, and N501Y affects neither antibody.

fluorescence-activated cell sorting (FACS) to enrich functional RBD mutants that escape antibody binding (Figure S1), and using deep sequencing to quantify the extent to which each mutation is enriched in the antibody-escape population relative to the original population. The effect of each mutation is quantified by calculating its "escape fraction," which represents the fraction of yeast expressing this mutant that fall in the antibody-escape FACS bin (these fractions range from 0 for mutations with no effects to 1 for mutations that strongly escape antibody binding).

We used this approach to map how all RBD mutations affect binding by a recombinant form of LY-CoV555 and its 1:1 cocktail combination with recombinant LY-CoV016, and examined these maps alongside similar data[15] that we recently reported for LY-CoV016 alone (Figures 1A and S1; Data S1; interactive visualizations at https://jbloomlab.github.io/SARS-CoV-2-RBD_MAP_LY-CoV555/). The maps show that LY-CoV555 is escaped by mutations at a focused set of sites, with site E484 standing out as a hotspot of escape (Figure 1A). We layered onto the escape maps our previous deep mutational scanning measurements[20] of how mutations affect angiotensin I-converting enzyme 2 (ACE2) binding (Figure 1A) or expression of folded RBD (Figure S2) and found that mutations escaping LY-CoV555 often have no adverse effect on these 2 functional properties of the RBD.

Comparison of the LY-CoV555 escape map with a map we previously reported for LY-CoV016 shows that the latter antibody is primarily escaped by mutations at sites where mutations do not affect LY-CoV555 (e.g., K417 and N460; Figures 1A and S1). However, there are some sites where single mutations escape binding by both LY-CoV555 and LY-CoV016, and as a result a 1:1 cocktail of the 2 antibodies is escaped by several single mutations, including I472D, G485P, and Q493R/K (Figures 1A and S2; see the magnifiable interactive maps at https://jbloomlab.github.io/SARS-CoV-2-RBD_MAP_LY-CoV555/ to examine these mutations at higher resolution). Note that some of the other smaller cocktail escape mutations in the cocktail maps may reflect a higher potency of LY-CoV555 in the 1:1 cocktail rather than representing mutations that truly escape binding by both antibodies.

CellPress
OPEN ACCESS

A

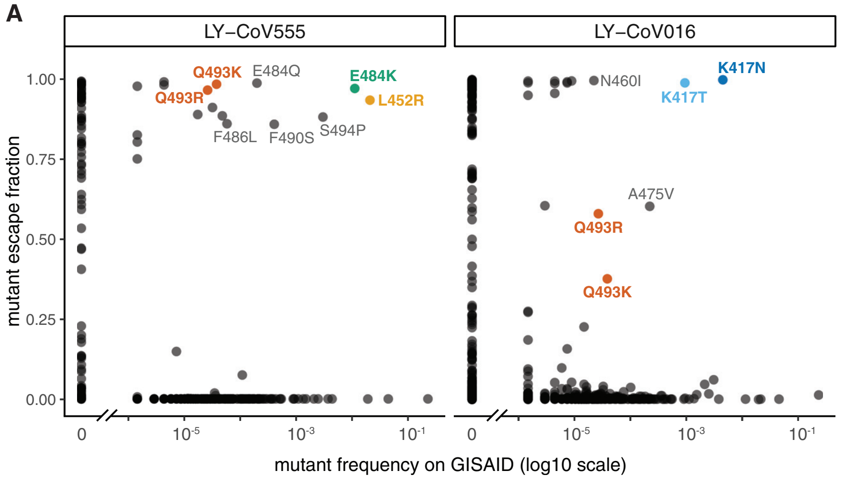

B

lineage	LY-CoV555 escape	LY-CoV016 escape	other RBD mutations
B.1.1.7 20I/501Y.V1 (UK)	none	none	N501Y
B.1.351 20H/501Y.V2 (S. Africa)	E484K	K417N	N501Y
P.1 20J/501Y.V3 (Brazil)	E484K	K417T	N501Y
B.1.429 20C/CAL.20C (USA)	L452R	none	none

Figure 2. Mutations present in sequenced SARS-CoV-2 isolates that escape antibody binding

(A) For each mutation, the escape fraction measured in the current (LY-CoV555) or prior (LY-CoV016[15]) study is plotted against the frequency of the mutation among all 679,454 high-quality human-derived SARS-CoV-2 sequences in GISAID as of March 15, 2021. Mutations with notable frequencies are labeled, and those discussed in the text are colored to key with (B) or to highlight observed cocktail escape mutations (Q493K/R).

(B) The RBD mutations in 4 emerging viral lineages, categorized by their effect on binding by LY-CoV555 and LY-CoV016. The B.1.351 and P.1 lineages contain combinations of mutations that escape each component of the LY-CoV555+LY-CoV016 cocktail. Lineages are described in the following references: B.1.1.7,[11] B.1.351,[10] P.1,[12] and B.1.429.[13]

Mutations at position Q493 are notably well tolerated with respect to ACE2 binding and RBD expression (Figures 1A and S2)—Q493K has been observed in a persistently infected immunocompromised patient.[15,21]

The binding measurements in our maps are consistent with previously reported effects of mutations on antibody neutralization from the literature (Figure 1B). Specifically, Wang et al.[14] have reported that E484K and K417N dramatically and specifically reduce neutralization by LY-CoV555 and LY-CoV016, respectively, while N501Y has no impact on neutralization by either antibody. However, our maps greatly extend this prior knowledge by identifying all of the mutations at all of the positions that affect binding by these antibodies and their combination.

We used the maps to assess how all of the RBD mutations present in sequenced SARS-CoV-2 isolates affect binding by each antibody (Figure 2A). The escape mutations present at the highest frequency among the sequenced isolates are E484K, L452R, and S494P for LY-CoV555 and K417N/T for LY-CoV016. An array of other mutations that escape each antibody are present at lower frequency among the sequenced isolates. Of particular note, the B.1.351 (also known as 20H/501Y.V2)[10] and P.1 (also known as 20J/501Y.V3)[12] lineages contain combinations of mutations (E484K and K417N/T) that individually escape each antibody (Figure 2B), suggesting that the LY-CoV555+LY-CoV016 cocktail may be ineffective against these lineages. In addition, the B.1.429 lineage (also known as 20C/CAL.20C) that has risen to high frequency in southern California contains L452R[13], which escapes LY-CoV555 (Figure 2B). Subsequent to the release of our original preprint version of this article, the US Food and Drug Administration's[22] (FDA's) fact sheet for bamlanivimab EUA was updated, confirming our findings by noting that L452R reduces bamlanivimab neutralization >1,000-fold. This observation coincides with recommendations to reduce the use of bamlanivimab monotherapy in locations where L452R is prominent. We also note that single mutations that escape both antibodies (Q493R and Q493K) have been observed in a handful of sequenced isolates (Figure 2A).

To gain insight into the structural basis for the escape mutations, we projected our escape maps onto crystal structures of the antibodies bound to the RBD[4,23] (Figure 3, interactive visualizations at https://jbloomlab.github.io/SARS-CoV-2-RBD_MAP_LY-CoV555/). LY-CoV016 and LY-CoV555 bind opposite sides of the receptor-binding ridge, a structurally[24] and evolutionarily[25,26] dynamic region of the RBD that forms part of the ACE2 receptor contact surface. The hotspots of escape for each antibody map closely to the core of each antibody-RBD complex. The sites where mutations escape the LY-CoV555+LY-CoV016 cocktail highlight their joint recognition of the receptor-binding ridge (Figure 3). The cocktail escape site Q493 is not in the receptor-binding ridge, but it is in a region of joint structural overlap by the 2 antibodies, such that the introduction of bulky, positively charged residues (R, K) may directly affect binding by each antibody.

DISCUSSION

We generated complete maps of mutations that escape a leading antibody and antibody cocktail being used to combat COVID-19. Our maps highlight the need to consider circulating SARS-CoV-2 diversity in regions where these antibodies are deployed, as several viral lineages already have mutations that escape binding from LY-CoV555 and its cocktail with LY-CoV016. The maps we report will continue to enable the immediate assessment of the effects of newly observed mutants on

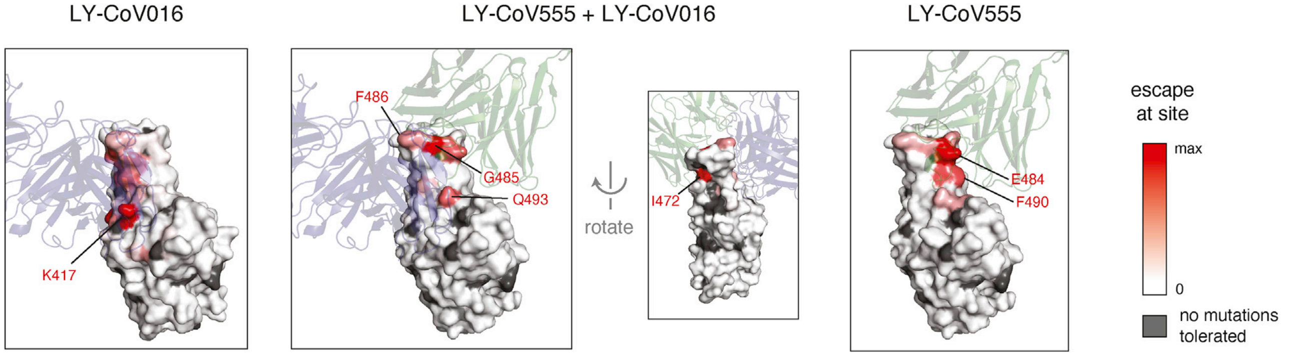

Figure 3. Escape maps projected onto structures of the RBD bound by LY-CoV555 or LY-CoV016

In each structure, the RBD surface is colored by escape at each site (white, no escape; red, strongest site-total escape for antibodies or strongest per-mutation escape for cocktail; gray, no escape because no mutations functionally tolerated). Sites of interest in each structure are labeled. The structures are as follows: LY-CoV016 (PDB: 7C01 [23]); LY-CoV555 (PDB: 7KMG [4]); cocktail escape projected onto the 7KMG structure, with the LY-CoV016 Fab chain aligned from the 7C01 structure for reference.

these antibodies and their cocktail, although it will of course remain necessary to validate key findings with additional virological experiments.

More broadly, our maps suggest that it may be advisable to more systematically consider possible escape mutations when devising antibodies for clinical use against SARS-CoV-2. It is now clear that human coronaviruses undergo antigenic evolution in response to immune pressure,[27,28] and we and others have begun to map out the key sites in the RBD that are targeted by human antibody immunity.[19,29–31] The recent increase in the frequency of mutations at site E484 suggests that this immunity may be beginning to drive antigenic variation within immunodominant positions in the RBD. Unfortunately, many of the leading therapeutic antibodies target the same epitopes as polyclonal antibody immunity, such as residue E484 or the 443-450 loop.[19] Because the clinical usage of monoclonal antibodies is unlikely to be so widespread as to drive viral evolution in the same way as infection- or vaccine-induced immunity, development of antibodies targeting less immunodominant epitopes may prove to be a strategy that is more resilient to the evolution of SARS-CoV-2.

Limitations of study

Our approach maps escape from antibody binding, but we do not directly measure the effects of mutations on *in vitro* neutralization or therapeutic protection. We note that our antibody binding-escape maps generated in prior studies have proven highly concordant with the effects of mutations on the antibody neutralization of mutant spike-pseudotyped viral particles.[15,18,32,33] Importantly, the conclusions of our work that are the most immediately relevant to clinical antibody use have been independently verified by pseudovirus neutralization assays. Specifically, as indicated in Figure 1B, Wang et al.[14] confirmed that E484K and K417N abolish neutralization by LY-CoV555 and LY-CoV016, respectively. After the posting of the original preprint version of our manuscript, the FDA[22] confirmed that L452R abolishes LY-CoV555 neutralization. Mutations that appear in our maps regularly incur $\geq$100-fold decreases in neutralization potency, although mutations with smaller effects on binding and neutralization can be below our sensitivity limit.[32] Our binding-escape maps are comprehensive, and therefore enable efficient and immediate prioritization of newly observed SARS-CoV-2 mutations for validation via antibody neutralization assays, tightening the feedback loop between genomic surveillance and functional evaluation.

STAR★METHODS

Detailed methods are provided in the online version of this paper and include the following:

- KEY RESOURCES TABLE
- RESOURCE AVAILABILITY
  - Lead contact
  - Materials availability
  - Data and code availability
- EXPERIMENTAL MODEL AND SUBJECT DETAILS
- METHOD DETAILS
  - Antibodies
  - Comprehensive profiling of mutations that escape antibody binding
  - Circulating variants
  - Data visualization
- QUANTIFICATION AND STATISTICAL ANALYSIS

SUPPLEMENTAL INFORMATION

Supplemental information can be found online at https://doi.org/10.1016/j.xcrm.2021.100255.

ACKNOWLEDGMENTS

We thank Bryan Jones and Eli Lilly & Company for sharing the LY-CoV555 sequence and structure, the Fred Hutch Flow Cytometry and Genomics facilities, and Fred Hutch Scientific Computing, supported by ORIP grant S10OD028685. This study was supported by the NIAID (R01AI127893 and R01AI141707, to J.D.B., and T32AI083203, to A.J.G.) and the Bill and Melinda Gates Foundation (INV-004949, to J.D.B.). T.N.S. is a Howard Hughes Medical Institute (HHMI) Fellow of the Damon Runyon Cancer Research Foundation (DRG-2381-19). J.D.B. is an Investigator of the HHMI.

AUTHOR CONTRIBUTIONS

All of the authors designed the study. T.N.S. performed the experiments and analyzed the data. T.N.S. and J.D.B. wrote the initial draft, and all of the authors edited the final version.

DECLARATION OF INTERESTS

The authors declare no competing interests.

Received: February 22, 2021
Revised: March 22, 2021
Accepted: March 26, 2021
Published: April 5, 2021

REFERENCES

1. Chen, P., Nirula, A., Heller, B., Gottlieb, R.L., Boscia, J., Morris, J., Huhn, G., Cardona, J., Mocherla, B., Stosor, V., et al.; BLAZE-1 Investigators (2021). SARS-CoV-2 Neutralizing Antibody LY-CoV555 in Outpatients with Covid-19. N. Engl. J. Med. *384*, 229–237.

2. Weinreich, D.M., Sivapalasingam, S., Norton, T., Ali, S., Gao, H., Bhore, R., Musser, B.J., Soo, Y., Rofail, D., Im, J., et al.; Trial Investigators (2021). REGN-COV2, a Neutralizing Antibody Cocktail, in Outpatients with Covid-19. N. Engl. J. Med. *384*, 238–251.

3. Tortorici, M.A., Beltramello, M., Lempp, F.A., Pinto, D., Dang, H.V., Rosen, L.E., McCallum, M., Bowen, J., Minola, A., Jaconi, S., et al. (2020). Ultrapotent human antibodies protect against SARS-CoV-2 challenge via multiple mechanisms. Science *370*, 950–957.

4. Jones, B.E., Brown-Augsburger, P.L., Corbett, K.S., Westendorf, K., Davies, J., Cujec, T.P., Wiethoff, C.M., Blackbourne, J.L., Heinz, B.A., Foster, D., et al. (2020). LY-CoV555, a rapidly isolated potent neutralizing antibody, provides protection in a non-human primate model of SARS-CoV-2 infection. bioRxiv. https://doi.org/10.1101/2020.09.30.318972.

5. Zost, S.J., Gilchuk, P., Case, J.B., Binshtein, E., Chen, R.E., Nkolola, J.P., Schäfer, A., Reidy, J.X., Trivette, A., Nargi, R.S., et al. (2020). Potently neutralizing and protective human antibodies against SARS-CoV-2. Nature *584*, 443–449.

6. Hassan, A.O., Case, J.B., Winkler, E.S., Thackray, L.B., Kafai, N.M., Bailey, A.L., McCune, B.T., Fox, J.M., Chen, R.E., Alsoussi, W.B., et al. (2020). A SARS-CoV-2 Infection Model in Mice Demonstrates Protection by Neutralizing Antibodies. Cell *182*, 744–753.e4.

7. Rogers, T.F., Zhao, F., Huang, D., Beutler, N., Burns, A., He, W.-T., Limbo, O., Smith, C., Song, G., Woehl, J., et al. (2020). Isolation of potent SARS-CoV-2 neutralizing antibodies and protection from disease in a small animal model. Science *369*, 956–963.

8. Eli Lilly & Company. Lilly's neutralizing antibody bamlanivimab (LY-CoV555) receives FDA emergency use authorization for the treatment of recently diagnosed COVID-19. https://investor.lilly.com/news-releases/news-release-details/lillys-neutralizing-antibody-bamlanivimab-ly-cov555-receives-fda.

9. Eli Lilly & Company. Lilly's bamlanivimab (LY-CoV555) administered with etesevimab (LY-CoV016) receives FDA emergency use authorization for COVID-19. https://investor.lilly.com/news-releases/news-release-details/lillys-bamlanivimab-ly-cov555-administered-etesevimab-ly-cov016.

10. Tegally, H., Wilkinson, E., Giovanetti, M., Iranzadeh, A., Fonseca, V., Giandhari, J., Doolabh, D., Pillay, S., San, E.J., Msomi, N., et al. (2021). Emergence of a SARS-CoV-2 variant of concern with mutations in spike glycoprotein. Nature. https://doi.org/10.1038/s41586-021-03402-9.

11. Public Health England (2020). Investigation of novel SARS-CoV-2 variant: Variant of Concern 202012/01.https://www.gov.uk/government/publications/investigation-of-novel-sars-cov-2-variant-variant-of-concern-20201201.

12. Faria, N.R., Mellan, T.A., Whittaker, C., Claro, I.M., da S. Candido, D., Mishra, S., Crispim, M.A.E., Sales, F.C., Hawryluk, I., McCrone, J.T., et al. (2021). Genomics and epidemiology of a novel SARS-CoV-2 lineage in Manaus, Brazil. medRxiv. https://doi.org/10.1101/2021.02.26.21252554.

13. Zhang, W., Davis, B.D., Chen, S.S., Sincuir Martinez, J.M., Plummer, J.T., and Vail, E. (2021). Emergence of a Novel SARS-CoV-2 Variant in Southern California. JAMA. https://doi.org/10.1001/jama.2021.1612.

14. Wang, P., Nair, M.S., Liu, L., Iketani, S., Luo, Y., Guo, Y., Wang, M., Yu, J., Zhang, B., Kwong, P.D., et al. (2021). Antibody Resistance of SARS-CoV-2 Variants B.1.351 and B.1.1.7. Nature. https://doi.org/10.1038/s41586-021-03398-2.

15. Starr, T.N., Greaney, A.J., Addetia, A., Hannon, W.W., Choudhary, M.C., Dingens, A.S., Li, J.Z., and Bloom, J.D. (2021). Prospective mapping of viral mutations that escape antibodies used to treat COVID-19. Science *371*, 850–854.

16. Hoffmann, M., Arora, P., Gross, R., Seidel, A., Hoernich, B., Hahn, A., Krueger, N., Graichen, L., Hofmann-Winkler, H., Kempf, A., et al. (2021). SARS-CoV-2 variants B.1.351 and B.1.1.248: escape from therapeutic antibodies and antibodies induced by infection and vaccination. bioRxiv. https://doi.org/10.1101/2021.02.11.430787.

17. Chen, R.E., Zhang, X., Case, J.B., Winkler, E.S., Liu, Y., VanBlargan, L.A., Liu, J., Errico, J.M., Xie, X., Suryadevara, N., et al. (2021). Resistance of SARS-CoV-2 variants to neutralization by monoclonal and serum-derived polyclonal antibodies. Nat. Med. https://doi.org/10.1038/s41591-021-01294-w.

18. Greaney, A.J., Starr, T.N., Gilchuk, P., Zost, S.J., Binshtein, E., Loes, A.N., Hilton, S.K., Huddleston, J., Eguia, R., Crawford, K.H.D., et al. (2021). Complete Mapping of Mutations to the SARS-CoV-2 Spike Receptor-Binding Domain that Escape Antibody Recognition. Cell Host Microbe *29*, 44–57.e9.

19. Greaney, A.J., Loes, A.N., Crawford, K.H.D., Starr, T.N., Malone, K.D., Chu, H.Y., and Bloom, J.D. (2021). Comprehensive mapping of mutations in the SARS-CoV-2 receptor-binding domain that affect recognition by polyclonal human plasma antibodies. Cell Host Microbe *29*, 463–476.e6.

20. Starr, T.N., Greaney, A.J., Hilton, S.K., Ellis, D., Crawford, K.H.D., Dingens, A.S., Navarro, M.J., Bowen, J.E., Tortorici, M.A., Walls, A.C., et al. (2020). Deep Mutational Scanning of SARS-CoV-2 Receptor Binding Domain Reveals Constraints on Folding and ACE2 Binding. Cell *182*, 1295–1310.e20.

21. Choi, B., Choudhary, M.C., Regan, J., Sparks, J.A., Padera, R.F., Qiu, X., Solomon, I.H., Kuo, H.-H., Boucau, J., Bowman, K., et al. (2020). Persistence and Evolution of SARS-CoV-2 in an Immunocompromised Host. N. Engl. J. Med. *383*, 2291–2293.

22. US Food and Drug Administration. FDA fact sheet for health care providers: emergency use authorization (EUA) of bamlanivimab. https://www.fda.gov/media/143603/download.

23. Shi, R., Shan, C., Duan, X., Chen, Z., Liu, P., Song, J., Song, T., Bi, X., Han, C., Wu, L., et al. (2020). A human neutralizing antibody targets the receptor-binding site of SARS-CoV-2. Nature *584*, 120–124.

24. Raghuvamsi, P.V., Tulsian, N.K., Samsudin, F., Qian, X., Purushotorman, K., Yue, G., Kozma, M.M., Hwa, W.Y., Lescar, J., Bond, P.J., et al. (2021). SARS-CoV-2 S protein:ACE2 interaction reveals novel allosteric targets. eLife *10*, e63646.

25. Guo, H., Hu, B.-J., Yang, X.-L., Zeng, L.-P., Li, B., Ouyang, S., and Shi, Z.-L. (2020). Evolutionary Arms Race between Virus and Host Drives Genetic Diversity in Bat Severe Acute Respiratory Syndrome-Related Coronavirus Spike Genes. J. Virol. *94*, e00902-20.

26. Shang, J., Ye, G., Shi, K., Wan, Y., Luo, C., Aihara, H., Geng, Q., Auerbach, A., and Li, F. (2020). Structural basis of receptor recognition by SARS-CoV-2. Nature *581*, 221–224.

27. Eguia, R., Crawford, K.H.D., Stevens-Ayers, T., Kelnhofer-Millevolte, L., Greninger, A.L., Englund, J.A., Boeckh, M.J., and Bloom, J.D. (2020). A human coronavirus evolves antigenically to escape antibody immunity. bioRxiv. https://doi.org/10.1101/2020.12.17.423313.

28. Kistler, K.E., and Bedford, T. (2021). Evidence for adaptive evolution in the receptor-binding domain of seasonal coronaviruses OC43 and 229e. eLife *10*, e64509.
29. Weisblum, Y., Schmidt, F., Zhang, F., DaSilva, J., Poston, D., Lorenzi, J.C.C., Muecksch, F., Rutkowska, M., Hoffmann, H.-H., Michailidis, E., et al. (2020). Escape from neutralizing antibodies by SARS-CoV-2 spike protein variants. eLife *9*, e61312.
30. Andreano, E., Piccini, G., Licastro, D., Casalino, L., Johnson, N.V., Paciello, I., Monego, S.D., Pantano, E., Manganaro, N., Manenti, A., et al. (2020). SARS-CoV-2 escape in vitro from a highly neutralizing COVID-19 convalescent plasma. bioRxiv. https://doi.org/10.1101/2020.12.28.424451.
31. Liu, Z., VanBlargan, L.A., Bloyet, L.-M., Rothlauf, P.W., Chen, R.E., Stumpf, S., Zhao, H., Errico, J.M., Theel, E.S., Liebeskind, M.J., et al. (2021). Identification of SARS-CoV-2 spike mutations that attenuate monoclonal and serum antibody neutralization. Cell Host Microbe *29*, 477–488.e4.
32. Greaney, A.J., Starr, T.N., Barnes, C.O., Weisblum, Y., Schmidt, F., Caskey, M., Gaebler, C., Cho, A., Agudelo, M., Finkin, S., et al. (2021). Mutational escape from the polyclonal antibody response to SARS-CoV-2 infection is largely shaped by a single class of antibodies. bioRxiv. https://doi.org/10.1101/2021.03.17.435863.
33. Dong, J., Zost, S.J., Greaney, A.J., Starr, T.N., Dingens, A.S., Chen, E.C., Chen, R.E., Case, J.B., Sutton, R.E., Gilchuk, P., et al. (2021). Genetic and structural basis for recognition of SARS-CoV-2 spike protein by a two-antibody cocktail. bioRxiv. https://doi.org/10.1101/2021.01.27.428529.
34. Wentz, A.E., and Shusta, E.V. (2007). A novel high-throughput screen reveals yeast genes that increase secretion of heterologous proteins. Appl. Environ. Microbiol. *73*, 1189–1198.
35. Otwinowski, J., McCandlish, D.M., and Plotkin, J.B. (2018). Inferring the shape of global epistasis. Proc. Natl. Acad. Sci. USA *115*, E7550–E7558.
36. Elbe, S., and Buckland-Merrett, G. (2017). Data, disease and diplomacy: GISAID's innovative contribution to global health. Glob. Chall. *1*, 33–46.
37. Katoh, K., and Standley, D.M. (2013). MAFFT multiple sequence alignment software version 7: improvements in performance and usability. Mol. Biol. Evol. *30*, 772–780.
38. Lan, J., Ge, J., Yu, J., Shan, S., Zhou, H., Fan, S., Zhang, Q., Shi, X., Wang, Q., Zhang, L., and Wang, X. (2020). Structure of the SARS-CoV-2 spike receptor-binding domain bound to the ACE2 receptor. Nature *581*, 215–220.
39. Hilton, S., Huddleston, J., Black, A., North, K., Dingens, A., Bedford, T., and Bloom, J. (2020). dms-view: Interactive visualization tool for deep mutational scanning data. J. Open Source Softw. *5*, 2353.

CellPress
OPEN ACCESS

STAR★METHODS

KEY RESOURCES TABLE

REAGENT or RESOURCE	SOURCE	IDENTIFIER
Antibodies		
FITC-conjugated chicken anti-cMyc antibody	Immunology Consultants Laboratory, Inc.	Cat# CMYC-45F
PE-conjugated goat anti-human-IgG	Jackson ImmunoResearch	Cat# 109-115-098
LY-CoV555 mAb	Genscript	Sequence from PDB: 7KMG
LY-CoV016 mAb	[15]	N/A
Deposited data		
Raw sequencing data	This paper	NCBI SRA: BioSample SAMN17836431
GISAID EpiCoV SARS-CoV-2 sequence isolates	GISAID	Full list of contributing labs and accessions: https://github.com/jbloomlab/SARS-CoV-2-RBD_MAP_LY-CoV555/blob/main/data/gisaid_hcov-19_acknowledgement_table_2021_03_15.pdf
Antibody-bound RBD structures	[4,23]	PDB: 7KMG, 7C01
Experimental models: cell lines		
Saccharomyces cerevisiae strain AWY101	[34]	AWY101
Oligonucleotides		
primers for Illumina sequencing amplicon generation	[20]	sequences given at https://github.com/jbloomlab/SARS-CoV-2-RBD_DMS/blob/master/data/primers/primers.csv
Recombinant DNA		
pETcon_SARS-CoV-2_RBD	Addgene	Plasmid ID: 166782
SARS-CoV-2 RBD mutant plasmid library	Addgene	Plasmid ID: 1000000172
Software and algorithms		
dms_variants, version 0.8.2	GitHub	https://jbloomlab.github.io/dms_variants/
Dmslogo	GitHub	https://jbloomlab.github.io/dmslogo/
custom code	This paper	all analyses provided on GitHub: https://github.com/jbloomlab/SARS-CoV-2-RBD_MAP_LY-CoV555

RESOURCE AVAILABILITY

Lead contact

Further information and requests for resources should be directed to and will be fulfilled by the Lead Contact, Jesse Bloom (jbloom@fredhutch.org)

Materials availability

SARS-CoV-2 mutant libraries and unmutated RBD plasmid have been deposited to Addgene, #166782 and #1000000172.

Data and code availability

- Complete computational pipeline: https://github.com/jbloomlab/SARS-CoV-2-RBD_MAP_LY-CoV555
- Markdown summaries of computational analysis: https://github.com/jbloomlab/SARS-CoV-2-RBD_MAP_LY-CoV555/blob/main/results/summary/summary.md
- Raw data table of mutant escape fractions: Data S1 and https://github.com/jbloomlab/SARS-CoV-2-RBD_MAP_LY-CoV555/blob/main/results/supp_data/LY_cocktail_raw_data.csv
- Raw Illumina sequencing data: NCBI SRA, BioProject: PRJNA639956, BioSample SAMN17836431

EXPERIMENTAL MODEL AND SUBJECT DETAILS

Saccharomyces cerevisiae strain AWY101[34] was cultured at 30°C in baffled flasks shaken at 275rpm for routine growth, or room temperature with mild agitation for induction of RBD surface expression. Selective media contained 6.7 g/L Yeast Nitrogen Base, 5.0 g/L Casamino acids, 1.065 g/L MES, and 2% w/v carbon source (dextrose for routine growth, galactose supplemented with 0.1% dextrose for RBD surface expression).

METHOD DETAILS

Antibodies

The LY-CoV555 antibody variable domain sequences were acquired from the LY-CoV555 crystal structure file (PDB: 7KMG [4]), which was generously shared by Bryan Jones and Eli Lilly and Co. prior to its publication. Purified antibody was produced by Genscript as human IgG in HD 293F mammalian cells, and affinity purified over RoboColumn Eshmuno A 0.6mL columns. LY-CoV016 was previously produced via the same approach, as described in Starr et al.[15]

Comprehensive profiling of mutations that escape antibody binding

Antibody escape mapping experiments were performed in biological duplicate using a deep mutational scanning approach. Assays were performed exactly as described by Starr et al.,[15] based on the approach first described in Greaney et al.[18] Briefly, yeast-surface display libraries expressing 3,804 of the 3,819 possible amino acid mutations in the SARS-CoV-2 RBD (Wuhan-Hu-1 sequence, GenBank MN908947, residues N331-T531) were previously sorted to select mutants capable of binding human ACE2. Libraries were induced for RBD surface expression and labeled with 400 ng/mL antibody (LY-CoV555, or 200 ng/mL each of LY-CoV555 and LY-CoV016 for 400 ng/mL total antibody). Cells were then incubated with 1:200 PE-conjugated goat anti-human-IgG (Jackson ImmunoResearch 109-115-098) to label for bound antibody and 1:100 FITC-conjugated anti-Myc (Immunology Consultants Lab CYMC-45F) to label for RBD surface expression. Yeast expressing the unmutated SARS-CoV-2 RBD were prepared in parallel to library samples and labeled at 400 ng/mL and 4 ng/mL with the corresponding antibody/cocktail for setting selection gates.

Antibody-escape cells were selected via fluorescence-activated cell sorting (FACS) on a BD FACSAria II. FACS selection gates (Figure S1) were drawn to capture 95% of unmutated yeast labeled at the 100x reduced 4 ng/mL antibody labeling concentration. For each sample, 10 million RBD+ cells were processed on the cytometer to sort out antibody-escape cells (fractions shown in Figure S1B), which were grown out overnight. Plasmid was purified from pre-sort and antibody-escape populations, and mutant frequencies pre- and post-sort were determined by Illumina sequencing of variant-identifier barcodes, exactly as described in Starr et al.[20]

Escape fractions were computed as described in Starr et al.[15] Briefly, we used the dms_variants package (https://jbloomlab.github.io/dms_variants/, version 0.8.2) to process Illumina sequences into counts of each barcoded RBD variant using the barcode/RBD look-up table from Starr et al.[20] The escape fraction of each library variant was determined as the fraction of cells carrying a particular barcode that were sorted into the antibody-escape bin, using the equation given in Greaney et al.[18] Scores were filtered for minimum library representation and mutant functionality as described in Starr et al.,[15] and single mutant escape scores were deconvolved using global epistasis models.[35] Mutation- and site-wise escape fractions correlated well between biological duplicates (Figure S1C), and we report the average of duplicate measurements in this paper. Raw values for mutation escape fractions given in Data S1. Markdown summaries of all steps of computational analysis are available on GitHub: https://github.com/jbloomlab/SARS-CoV-2-RBD_MAP_LY-CoV555/blob/main/results/summary/summary.md.

Circulating variants

All spike sequences present on GISAID[36] as of March 15, 2021 were downloaded and aligned via mafft.[37] Sequences from non-human origins, sequences with gaps or ambiguous characters, and sequences with more than 8 RBD mutations from consensus were removed. RBD amino acid differences were enumerated compared to the Wuhan-Hu-1 RBD sequence. We acknowledge all contributors to the GISAID EpiCoV database for their sharing of sequence data. (All contributors listed at: https://github.com/jbloomlab/SARS-CoV-2-RBD_MAP_LY-CoV555/blob/main/data/gisaid_hcov-19_acknowledgement_table_2021_03_15.pdf).

Data visualization

Static logoplots were created using dmslogo (https://jbloomlab.github.io/dmslogo/). Interactive visualizations of the escape maps and their projection onto the ACE2-bound (PDB: 6M0J [38]) and antibody-bound structures available at https://jbloomlab.github.io/SARS-CoV-2-RBD_MAP_LY-CoV555/ were created using dms-view (https://dms-view.github.io/docs/).[39] For Figure 3, escape scores were mapped to PDB b-factors and visualized in PyMol using antibody-bound RBD structures PDB 7KMG [4] and PDB 7C01.[23]

QUANTIFICATION AND STATISTICAL ANALYSIS

Experiments were conducted in full biological duplicate, using independently generated and assayed mutant libraries. Values used throughout the text are the average of these duplicate measurements, as described in Method details and Figure S1 legend.

Cell Reports Medicine, Volume *2*

Supplemental information

Complete map of SARS-CoV-2 RBD mutations that escape the monoclonal antibody LY-CoV555 and its cocktail with LY-CoV016

Tyler N. Starr, Allison J. Greaney, Adam S. Dingens, and Jesse D. Bloom

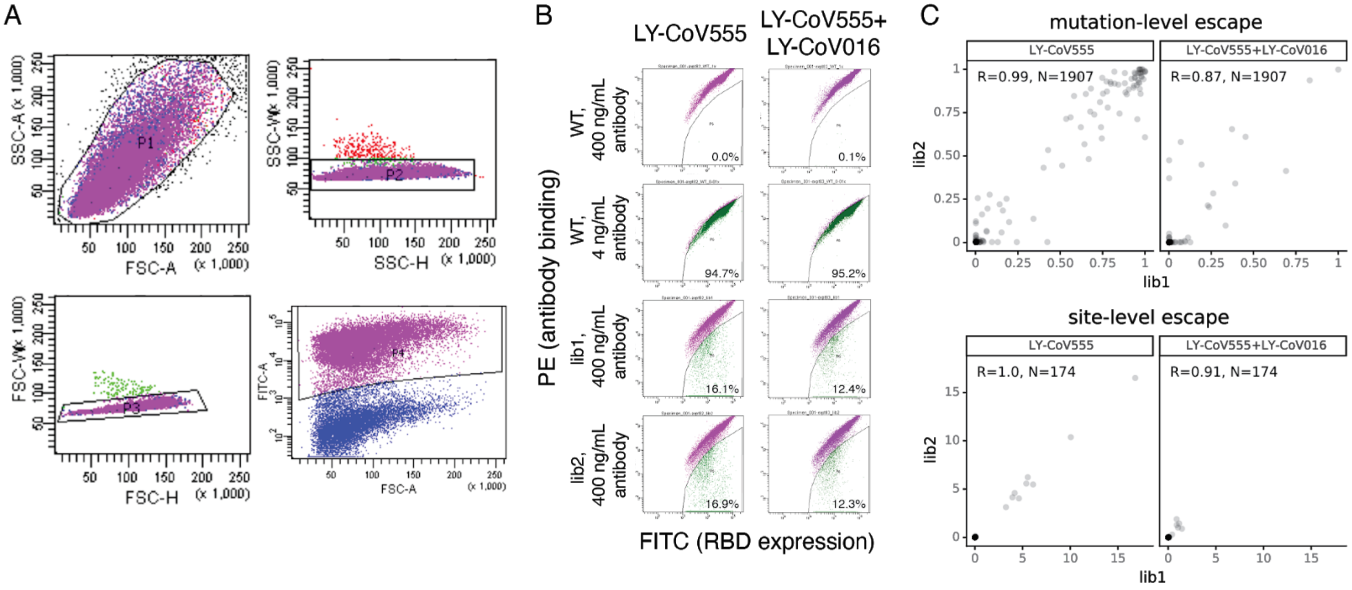

Figure S1. Experimental approach for antibody escape mapping. Related to Figure 1. We use fluorescence-activated cell sorting to select yeast mutants that escape antibody binding. We use libraries described by Starr et al. [S1] that contain virtually all RBD amino acid mutations, which were previously sorted to select for those compatible with human ACE2 binding as described by Greaney et al. [S2]. (A) Initial FACS gates are drawn to select single yeast cells (SSC/FSC, SSC-W/SSC-H, and FSC-H/FSC-W) that properly express RBD (FITC/FSC). (B) Antibody-escape bins are drawn to capture 95% of RBD^{+} cells expressing the unmutated SARS-CoV-2 RBD labeled at 4 ng/mL, indicating 100x reduced binding compared to the 400 ng/mL library selections. The fraction of cells in each population that fall into this “antibody-escape” bin is labeled in each FACS plot. (C) Correlation between independent library duplicates at the level of per-mutation escape fractions (top) or site-level total escape (sum of all mutations at site, bottom). We report the average of these duplicate measurements in the text, given in Data S1.

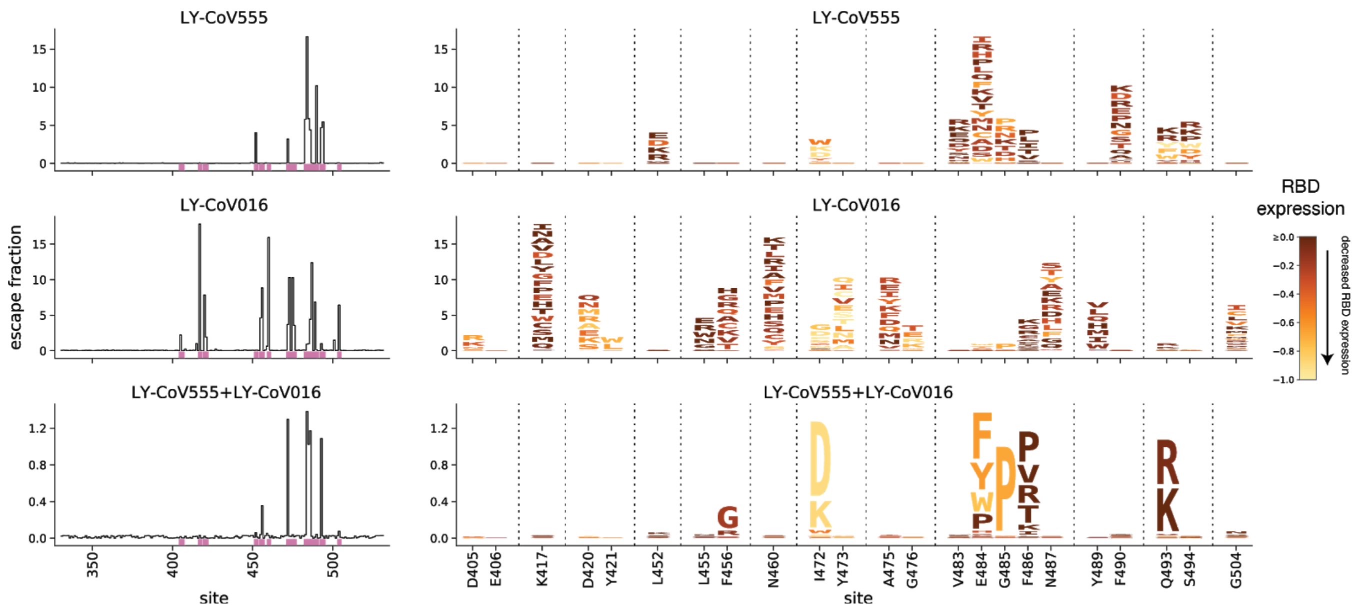

Figure S2. Logoplots colored by mutation effects on expression. Related to Figure 1. Line and logoplots are identical to Fig. 1A, except mutations are colored by their effects on the yeast surface expression level of folded RBD, as measured in our previous deep mutational scanning experiment [S1].

Supplemental References

[S1] Starr TN, Greaney AJ, Hilton SK, et al. Deep Mutational Scanning of SARS-CoV-2 Receptor Binding Domain Reveals Constraints on Folding and ACE2 Binding. *Cell* 2020; 182: 1295–1310.e20.

[S2] Greaney AJ, Starr TN, Gilchuk P, et al. Complete Mapping of Mutations to the SARS-CoV-2 Spike Receptor-Binding Domain that Escape Antibody Recognition. *Cell Host Microbe* 2021; 29: 44–57.e9.
